# Modelling large scale artery haemodynamics from the heart to the eye in response to simulated microgravity

**DOI:** 10.1038/s41526-024-00348-w

**Published:** 2024-01-13

**Authors:** Harrison T. Caddy, Lachlan J. Kelsey, Louis P. Parker, Daniel J. Green, Barry J. Doyle

**Affiliations:** 1 Vascular Engineering Laboratory, Harry Perkins Institute of Medical Research, Queen Elizabeth II Medical Centre, Nedlands, Australia and the UWA Centre for Medical Research, The University of Western Australia, Perth, WA Australia; 2 School of Human Sciences (Exercise and Sport Sciences), The University of Western Australia, Perth, WA Australia; 3 School of Engineering, The University of Western Australia, Perth, WA Australia; 4 FLOW, Department of Engineering Mechanics, KTH Royal Institute of Technology, Stockholm, Sweden

**Keywords:** Predictive markers, Biomedical engineering

## Abstract

We investigated variations in haemodynamics in response to simulated microgravity across a semi-subject-specific three-dimensional (3D) continuous arterial network connecting the heart to the eye using computational fluid dynamics (CFD) simulations. Using this model we simulated pulsatile blood flow in an upright Earth gravity case and a simulated microgravity case. Under simulated microgravity, regional time-averaged wall shear stress (TAWSS) increased and oscillatory shear index (OSI) decreased in upper body arteries, whilst the opposite was observed in the lower body. Between cases, uniform changes in TAWSS and OSI were found in the retina across diameters. This work demonstrates that 3D CFD simulations can be performed across continuously connected networks of small and large arteries. Simulated results exhibited similarities to low dimensional spaceflight simulations and measured data—specifically that blood flow and shear stress decrease towards the lower limbs and increase towards the cerebrovasculature and eyes in response to simulated microgravity, relative to an upright position in Earth gravity.

## Introduction

Fluid simulations enable the investigation of blood flow distribution in the cardiovascular system to better understand disease progression, inform surgical procedures and evaluate responses to internal and external conditions affecting the body. These simulations can also be used to reduce the risks associated with extreme environments, such as the microgravity experienced by astronauts during long-duration spaceflight where cardiovascular and muscular deconditioning can occur along with the development of a condition known as space associated neuro-ocular syndrome (SANS)^1^, impairing vision.

There are a number of cardiovascular-related changes that can arise during, or as a result of, long-duration spaceflight including large fluid shifts and stroke volume changes, variations in blood pressure, vascular tissue changes and orthostatic intolerance^2^. Terrestrially based research can be performed to emulate phenomena associated with spaceflight and to investigate the long-term implications on the body through methods such as long-duration head-down tilt (HDT) and water immersion experiments or parabolic flights. However, these experiments generally require extensive planning and are often associated with high costs due to duration and equipment requirements^3^. Furthermore, HDT experiments may not necessarily accurately emulate microgravity as they induce artificial pressure gradients, whilst parabolic flights can only provide short exposure windows of 20–30 s at a time^4^. Alternatively, computational fluid dynamics (CFD) simulations offer a relatively low-cost approach to model fluid changes associated with spaceflight, in either human or animal models. In addition, simulations are also advantageous in that they can use retrospective data and account for the varying sizes and scales of cardiovascular networks throughout the body.

Zero-dimensional (0D) lumped parameter modelling is often employed to model large-scale arterial networks across a wide range of conditions with relatively low computational cost. However, a drawback of low dimensional models is the inability to capture localised haemodynamics such as wall shear stress (WSS) distributions, or non-uniform flow through vessels due to geometric factors such as stenosis, bifurcations, tortuosity or high degrees of vessel curvature. Three-dimensional (3D) CFD simulations of the vasculature enable the evaluation of localised flow to a high degree of spatial and temporal resolution^5,6^. However, 3D CFD simulations often omit vascular networks upstream and downstream of a domain of interest due to increased computational cost. In place of an upstream geometry, inlet boundary conditions can be specified using measured data, existing literature or from 0D modelling. Downstream of a domain of interest outlet boundaries can be prescribed using zero pressure or specified flow split outlets. Alternatively, resistive elements, fractal trees or Windkessel modelling can be employed to emulate the downstream resistance and compliance of peripheral arteries and venous networks.

Lower dimensional fluid mechanics studies (i.e., 0D, 1D and 2D) have previously been used to model the arterial tree leading to the cerebrovasculature, or within the eye, in attempts to understand disease development such as glaucoma^7^, diabetic retinopathy^8^, hyper- and hypotension^9^ as well as the effects of spaceflight or ground-based HDT experiments^4,10^. Studies have simulated blood flow within large arterial networks for the purposes of understanding pulse wave velocity propagation and age-related arterial stiffening^11^, arterial particle and embolism transport^12,13^, calculation of the ankle-brachial index^14^, effect of venoarterial extracorporeal membrane oxygenation^15^ and demonstration of meshing strategies and computational optimisation^16^. Previous large-scale simulations have also encountered challenges in accurately evaluating localised haemodynamic metrics such as WSS due to computational limitations^11^. Development of a large-scale human artery haemodynamics framework enables verification with existing models of blood flow under conditions such as simulated microgravity^4,10,17^.

In this study, we aimed to construct a physiologically possible 3D model of human blood vessels ranging from the aortic root through to the retina, by combining existing subject-specific 3D models of arterial vessels from different sources. With this geometry, we developed fluid simulations that accommodated a single continuum physics model for modelling blood across a large arterial network, and gravitational effects to investigate the distributions of blood flow to distal small arteries, specifically in the eye. We used this framework to compare haemodynamic metrics at arterial regions of interest within this vessel network in response to Earth’s gravitational conditions and simulated microgravity.

## Methods

### Imaging methods and 3D reconstruction

**Fig. 1:**
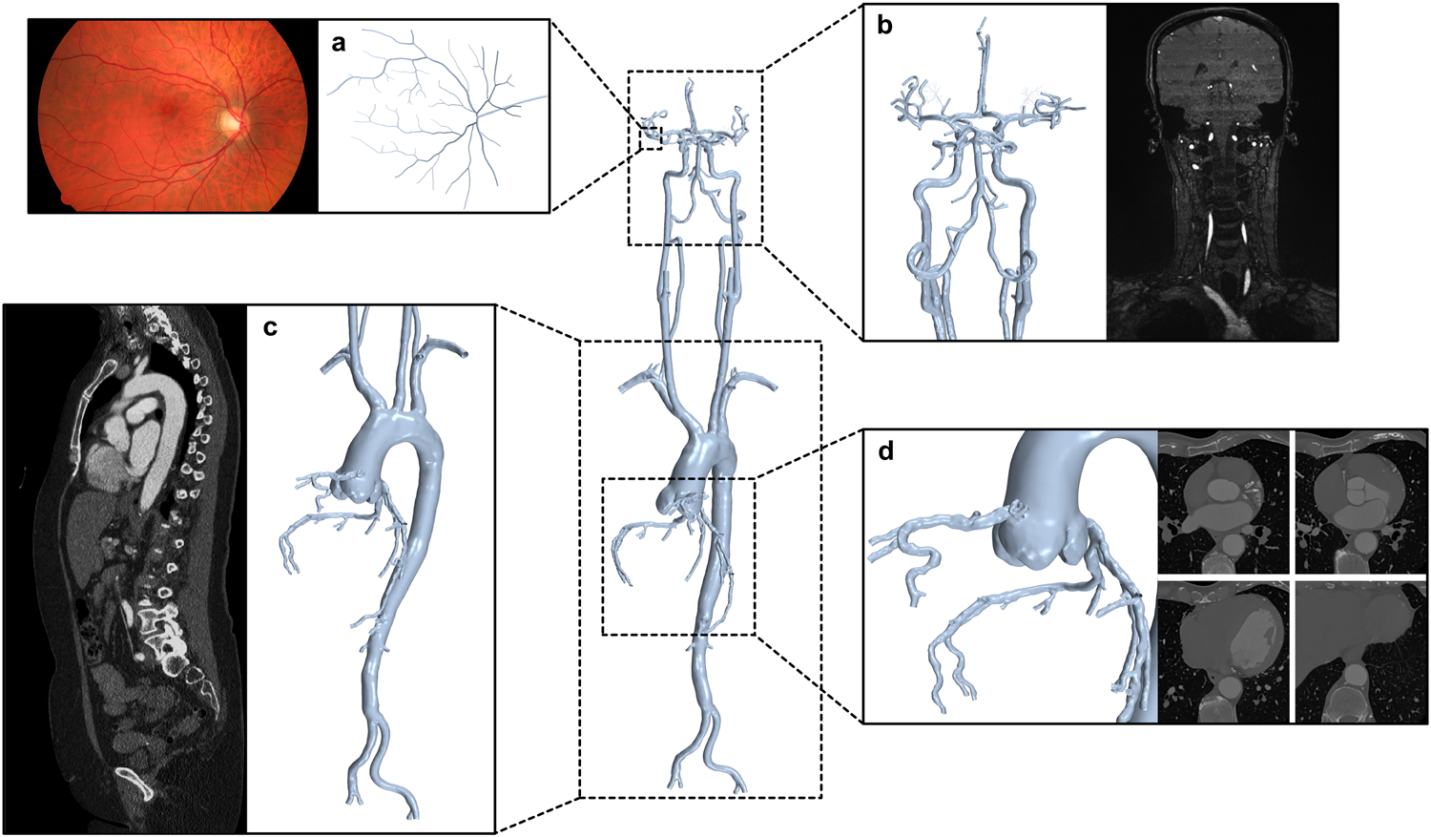
Continuous arterial network model. Examples of types of corresponding image data are provided alongside detailed views of the 3D geometry. This model was comprised of four 3D reconstructions associated with separate image datasets, consisting of the retinal arterioles (**a**) with corresponding image adapted from^23^ (CC BY 3.0), cerebrovasculature and neck arteries (**b**), aorta and iliac arteries (**c**) and the aortic root and coronary arteries (**d**). The 3D reconstructions of these regions were combined within STAR-CCM+.

This study was approved by the University of Western Australia (2022/ET000688). We sourced 3D arterial vasculature models from numerous past studies^18–22^. We imported the associated stereolithography files into STAR-CCM+ (v15, Siemens, Munich, Germany), where we used the surface repair tool to manually perform iterative rigid transformations to closely align each adjacent model using general imaging landmarks (e.g., aortic arch), combine selected overlapping vertices and then iteratively smooth the overlapping regions to achieve an average lumen (Fig. 1).

The superficial retinal arteriole section of the 3D model used data from a previous study^18^, which was reconstructed from a publicly available retinal fundus image of a healthy eye (CANON CF-60UVi) from the High-Resolution Fundus Image Database^23^. Briefly, the images were filtered and converted to binary processed images before manual segmentation of the arterioles and their diameter using open-source graphics editing software (GIMP, GIMP Team, California, United States). The resulting image was then segmented again in Mimics (v18, Materialise, Belgium) to create a 3D geometry and the centerline was extracted using 3-Matic (v10, Materialise, Belgium), before creating lofted cylinders along these centerlines and transforming the 3D model to a spherical curvature with a radius of 11.824 mm using a coordinate transformation in MATLAB (2016b, Mathworks, Massachusetts, United States). This model was duplicated and applied for both the left and right eyes, correcting for nasal and temporal orientation on either side. More detail on the image analysis and geometry creation is provided in Rebhan et al.^18^.

The 3D model of the cerebrovascular and neck arteries was obtained as part of a previous study^19^, where participants were imaged using 3 T time-of-flight magnetic resonance angiography (3 T TOF MRA) (Siemens Magnetom, Skyra) with a corresponding pixel size of 0.31 mm and a slice thickness of 0.75 mm. These images were reconstructed using in-house software to create a 3D isosurface, which was subsequently smoothed to within 5% of its starting volume and reconstruction artefacts were removed. While segmenting the cerebrovascular and neck arteries, a manual mask segmentation was created that followed the centre of the optic nerve sheath from the retina to the ophthalmic artery, representing the branching central retinal artery (CRA). This segmented cylindrical line was initially at the resolution of the 3 T TOF MRA images at approximately 0.3 mm in diameter. Upon importation into STAR-CCM+ for surface repair, we reduced the thickness of this line to 163 μm using local mesh smoothing techniques to match the average measured diameter of the CRA in healthy individuals^24–26^.

The 3D model of the aortic root and coronary arteries was obtained from previously reconstructed computed tomography (CT) coronary angiogram images from a recent study^20,21^ which had a pixel size of 0.45 mm and slice thickness of 0.8 mm in Mimics. The aorta, iliac and femoral artery 3D model network was obtained from reconstructed and modified imaging data from a previous study^22^, which used continuous arterial phase CT imaging with a slice thickness and increment size of 2.5 mm and 1 mm, respectively.

### Computational fluid dynamics

Simulations were developed in the commercial CFD package STAR-CCM+. In this study, we created two investigation cases; Earth gravity and simulated microgravity which both used the same 3D arterial geometry.

We used a combination of a trimmer cell mesh in the core of the fluid geometry and prescribed anisotropic prism layer cells to capture the behaviour of the fluid at the near-wall boundary. These prism layer cells were distributed in both the small and large arterial vessels with variable thickness to the regions of the retina, cerebrovasculature, neck and coronary arteries using volumetric controls. To ensure mesh independence we used the non-uniform refinement ratio formulation of the grid convergence index (GCI)^27,28^ across a range of different haemodynamic parameters—with calculated GCI values for mass flow rate and WSS metrics falling below 2-3% indicating sufficient mesh discretisation^18,19,29^. Mesh sizes can be found in Supplementary Table 1, settings in Supplementary Table 2 and GCI results in Supplementary Table 3. The final mesh consisted of ~43 million elements.

Blood was modelled as an incompressible fluid with a density of 1050 kg m^-3^ ^30^. We assumed rigid walls with a no-slip boundary condition and a laminar flow regime, as this is expected in the majority of the fluid domain under normal healthy conditions. To capture the variation in blood viscosity due to both the non-Newtonian shear thinning nature as well as a reduction in viscosity due to the Fåhraeus-Lindqvist (FL) effect, a blended viscosity model was implemented. Using wall distance, we determined vessel diameter and prescribed the FL viscosity model^30,31^ below vessel diameters of 0.6 mm^32^, the Carreau-Yasuda model as described by Karimi et al.^33^ (*η*_∞_ = 0.0035 Pa s; *η*_0_ = 0.16 Pa s; *λ* = 8.2 s; *a* = 0.64; *n* = 0.2128) in vessels greater than 1.2 mm, and linearly interpolated between these two models within this diameter range (0.6–1.2 mm).

For the gravity case, we used the mass flow waveform from Brown et al.^34^, which was prescribed in terms of a parabolic velocity profile at the aortic root. For each of the retinal arteriole outlets, we calculated outlet resistances using a structured asymmetrical fractal tree specific for retinal arteries as described by Malek et al.^31^, which is an extension of methods developed by those such as Olufsen^35^. Briefly, this method describes the branching of the daughter vessel radius from the parent vessel radius in terms of an exponent law and an asymmetry index, which allows for asymmetrical weighting of vessel branching. To calculate the resistances associated with a fractal tree network, a length ratio was assumed that varied depending on the branching vessel diameter. Vessel outlets that branched to a diameter below an assumed retinal capillary bed diameter of 4 μm^36^ were assumed to have a pressure of 0 mmHg. The non-Newtonian behaviour of blood viscosity within small arterioles due to the FL effect is known to substantially affect upstream haemodynamics^37^. Consequently, we used an implementation of the FL viscosity model described by Liu et al.^30^, assuming a haematocrit value of 0.45, a plasma viscosity of 1.2 mPa s and blood density of 1050 kg m^-3^ ^30^. Resistance values calculated for each retinal arteriole outlet were then converted to a corresponding effective viscosity value using the Hagen-Poiseuille equation for incompressible laminar fluid flow within cylindrical pipes (Eq. 1).

1 $${\mu }_{i}=\frac{{R}_{i}{\rm{\pi }}{r}_{i}^{4}}{8{L}_{i}}$$

Where, for outlet *i*, *µ*_*i*_ is the effective viscosity, *R*_*i*_ is the fractal tree calculated resistance, and *r*_*i*_ and *L*_*i*_ are the radius and length respectively.

We then implemented each representative resistance effective viscosity value within a corresponding extruded outlet region specific for each arteriole. The extrusion length was set as double the outlet diameter and the distal surface was prescribed a zero-pressure condition, as per the assumption of prescribing a pressure of 0 mmHg at the capillary bed.

For each of the remaining arterial outlets outside of the retinal arterioles, we calculated the desired resistance for each outlet, which we then converted into a viscosity term using the same Hagen-Poiseuille Eq. (1) and applied this within an extruded outlet region. To do this, we assumed a pressure drop from systolic pressure to zero across each extruded outlet region (representing the pressure drop towards distal capillary beds) and used the corresponding systolic volume flow rate from the pressure and inlet waveforms respectively from Brown et al.^34^. The volumetric flow to each outlet was then scaled primarily by the percentage distribution of cardiac output to an overarching arterial region, and then secondarily using the corresponding outlet radius relative to the other outlet radii within the same arterial region using Murray’s law (Eq. 2). For the percentage of cardiac output to each region, we obtained values from literature of the estimated percentage of cardiac output to different arterial regions, which are summarised in Table 1. Flow to the subclavian arteries was calculated from the residual of total cardiac output and paired arterial regions were assumed to have symmetrical distribution of flow to each.

2 $${R}_{i}=\frac{{P}_{{\rm{sys}}}}{{Q}_{{\rm{sys}}}\times {\rm{C}}{{\rm{O}}}_{{\rm{split}}}\times \frac{{r}_{i}^{3}}{\mathop{\sum }\nolimits_{j=1}^{N}{r}_{j}^{3}}}$$

Where, for outlet *i*, *R*_*i*_ is the calculated resistance, *P*_sys_ and *Q*_sys_ are the systolic pressure and flow values from Brown et al.^34^, CO_split_ is the estimated percentage split of cardiac output to an arterial region as summarised in Table 1, *r*_*i*_ is the outlet radius and *N* is the number of outlets in a corresponding arterial region.

**Table 1:** Assumed flow distribution to different arterial networks expressed as a percentage of cardiac output.

Artery region (*N*)	Cardiac output	Refs.
Celiac artery (1)	14.00%	^99^
Cerebral arteries (32)	12.78%	^100^
Coronary arteries (26)	5.00%	^101^
External carotid artery (2)	9.45%	^100^
Internal iliac artery (2)	4.00%	^99^
External iliac artery (2)	9.00%	^99^
Ophthalmic artery (2)	0.68%	^102^
Mesenteric artery (1)	16.00%	^99^
Renal artery (2)	23.00%	^99^
Subclavian artery (2)	6.09%	-

The number of vessels associated with each arterial network is provided in brackets (*N*). Paired arteries are assumed to have symmetrical flow distribution to each side. Flow to the subclavian arteries was assumed to be the residual of cardiac output.

Each resistance outlet viscosity term was then implemented within a corresponding extruded outlet region, which was prescribed a length twice the outlet diameter and a zero-pressure boundary condition was imposed at the distal extrusion surface. For the gravity case, we prescribed the typical Earth gravitational acceleration of 9.81 m s^–2^ ^38^ (1 g) acting inferiorly to emulate an upright position.

For the simulated microgravity case, we modified the inlet waveform from Brown et al.^34^ to account for the effects observed during spaceflight. Cardiac output is generally reported to increase in response to microgravity, with documented increases of 10%^39^, 20%^40,41^ as well as up to 30-40%^42^, and even in excess of 50%^1,43^. Heart rate is known to be relatively constant during spaceflight^44^, if not slightly decreased^45^, indicating that there is an increase in stroke volume. To model this change, we vertically scaled the waveform from Brown et al. until the stroke volume (and hence cardiac output) was increased by an assumed 20%^46^ with the aim to emulate a moderate increase in pre-load from increased venous return as observed during spaceflight^42,46^. Furthermore, arterial resistance of the external iliac artery outlets (from Table 1) was increased by 93% to emulate the increased lower limb vascular resistance observed during spaceflight^47^ relative to an upright position, while all other outlets remained at the corresponding Earth gravity resistances, as few other arterial networks have observed changes in resistance in response to microgravity^48^. Finally, we set the gravitational acceleration to 0 m s^-2^.

### Simulation execution

All simulations were solved using the finite-volume method within STAR-CCM+. We used the segregated flow solver and the implicit unsteady model with second-order temporal discretization, which uses the Semi-Implicit Method for Pressure-Linked Equations (SIMPLE) algorithm for coupling pressure and velocity. We used a time step of 0.001 s and inner iterations were terminated if normalised momentum and continuity residuals fell below 10^-4^. Simulations were run for 3 cardiac cycles, whereupon data was extracted over a final fourth cycle, which was sufficient for the difference in cycle averaged metrics (i.e., global WSS, ICA and VA flow, etc.) to remain below 2–3% between the final two cycles for both the control and simulated microgravity cases. Simulations were run on Magnus, a Cray XC40 supercomputer (Pawsey Supercomputing Centre, Perth, Australia) using 600 cores across 25 compute nodes, each providing 24 cores per node. Simulations required approximately 20,500 core hours to complete, equating to 34 h of run time.

### Data extraction

For each case, we extracted mass flow rates leading to the cerebrovasculature and retina, maximal velocity waveforms within the CRA and M1 segments of the middle cerebral artery (MCA) and surface averaged time-averaged WSS (TAWSS), and oscillatory shear index (OSI) within the retina, Circle of Willis (CoW), carotid bifurcations, coronary and iliac arteries as well as within the ascending and descending aorta. Surface averaged data is presented as surface average ± surface standard deviation.

## Results

### Haemodynamic responses to simulated microgravity

**Fig. 2:**
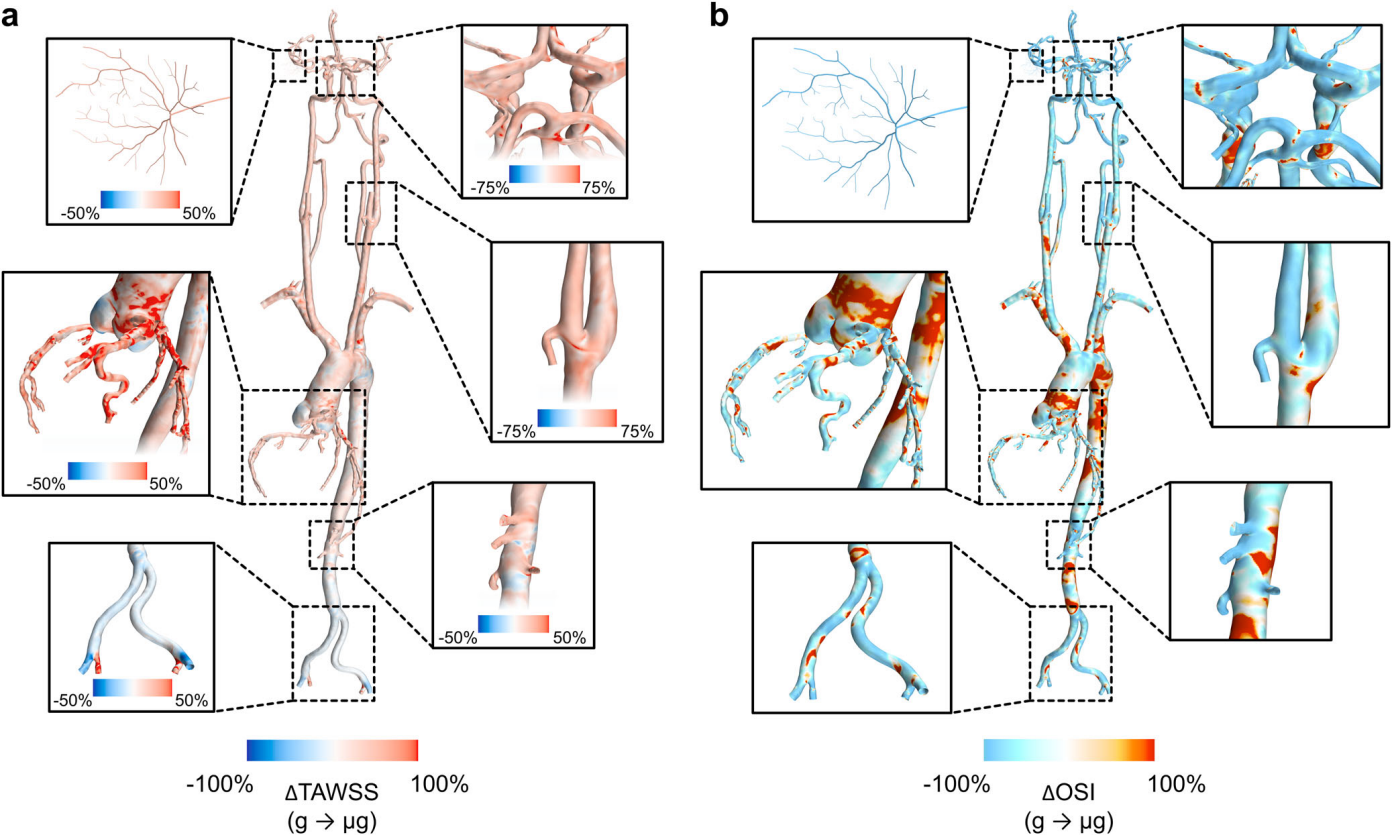
Entire arterial network surface distributions of haemodynamic metrics. Distributions show the relative change (%) in time-averaged wall shear stress (TAWSS) (**a**) and oscillatory shear index (OSI) (**b**) from Earth gravity to simulated microgravity (g→µg). Detail views (with varying colour bar scales for TAWSS) of the retinal, cerebrovascular, carotid bifurcation, coronary, branching visceral and renal, and iliac arteries are presented.

**Fig. 3:**
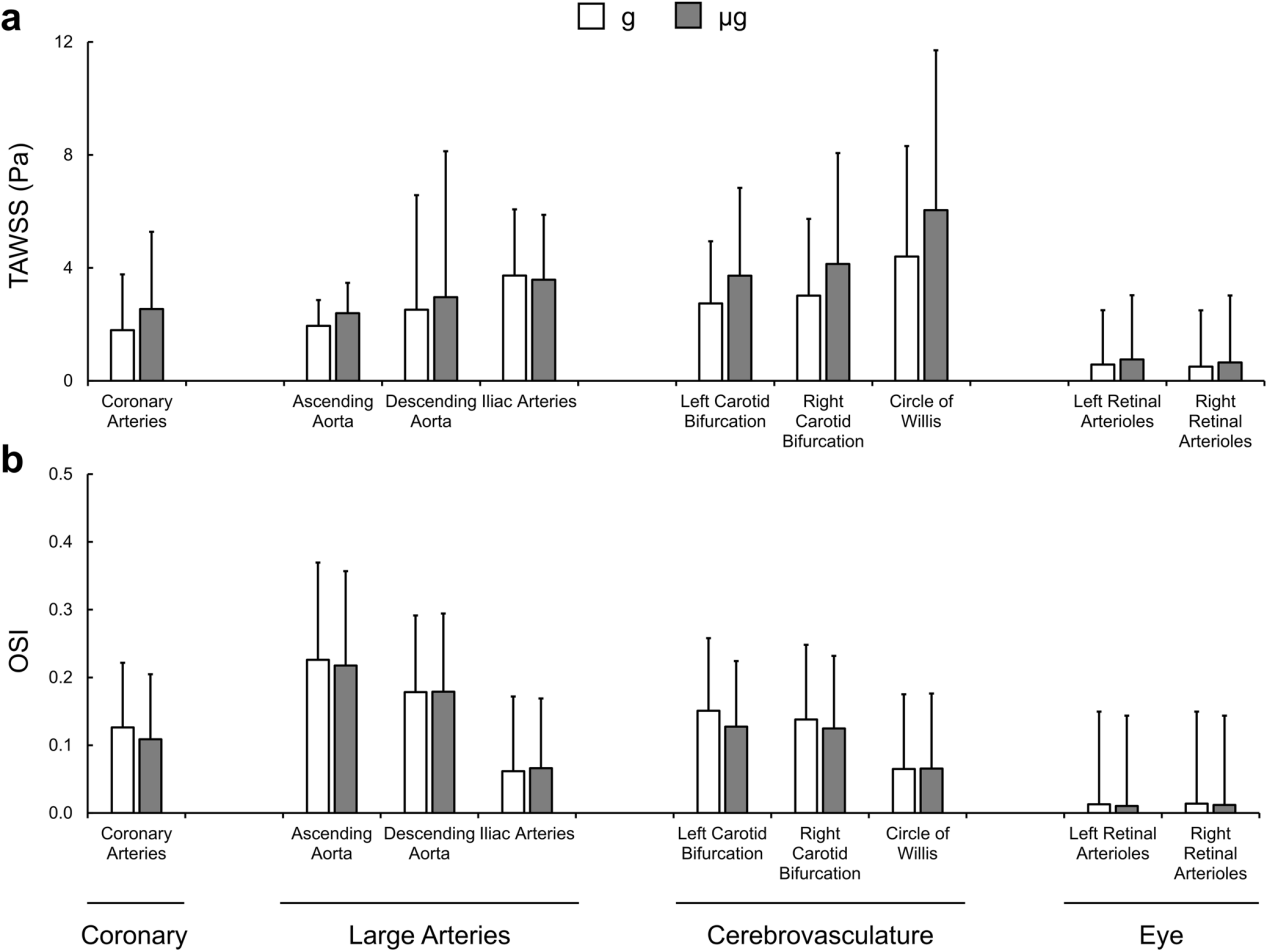
Surface averaged haemodynamic metrics. Data is presented for the Earth gravity (g; white) and simulated microgravity (µg; grey) cases. Measures of time-averaged wall shear stress (TAWSS) (**a**) and oscillatory shear index (OSI) (**b**) are presented as a surface average with error bars representing the corresponding regional surface standard deviation.

Qualitative distributions of the relative change in TAWSS and OSI between control and simulated microgravity conditions across the continuous arterial geometry as well as detailed views of regions of interest can be seen in Fig. 2. We extracted the surface averaged haemodynamic metrics across regions of interest throughout the entire 3D geometry (Fig. 3). Across both cases, absolute TAWSS was greatest in the CoW. The most substantial differences in TAWSS between gravity and simulated microgravity cases were found in the coronary arteries with increases of 41% (2.54 ± 2.74 Pa vs. 1.80 ± 1.97 Pa), in the left and right carotid bifurcations with increases of 36–37% (left, 3.72 ± 3.11 Pa vs. 2.74 ± 2.21 Pa; right, 4.14 ± 3.93 Pa vs. 3.02 ± 2.72 Pa), in the CoW with increases of 37% (6.04 ± 5.66 Pa vs. 4.40 ± 3.91 Pa) and within the left and right retinal arterioles with increases of 29-31% (left, 0.76 ± 2.27 Pa vs. 0.58 ± 1.92 Pa; right, 0.65 ± 2.37 Pa vs. 0.50 ± 1.99 Pa). Less substantial increases of 23% in the ascending (2.39 ± 1.08 Pa vs. 1.95 ± 0.91 Pa) and 17% in the descending (2.96 ± 5.17 Pa vs. 2.52 ± 4.05 Pa) aorta were found. In comparison, the TAWSS in the iliac arteries decreased by 4% (3.58 ± 2.30 Pa vs. 3.73 ± 2.35 Pa). Across both cases, absolute OSI was greatest in the ascending and descending aorta. In general, we observed a decrease in surface averaged distributions of OSI between gravity and simulated microgravity cases, with the largest decreases of –19% and –14%, respectively, in the left and right retinal arterioles (left, 0.010 ± 0.133 vs. 0.013 ± 0.137; right, 0.012 ± 0.132 vs. 0.014 ± 0.136), –16% and –10%, respectively, in the left and right carotid bifurcations (left, 0.127 ± 0.097 vs. 0.151 ± 0.107; right, 0.125 ± 0.107 vs. 0.138 ± 0.110) and –14% in the coronary arteries (0.109 ± 0.096 vs. 0.126 ± 0.096). Conversely, OSI in the descending aorta and CoW remained unchanged (0–1%), while a 7% increase was observed in the iliac arteries (0.066 ± 0.103 vs. 0.062 ± 0.110).

### Head and neck artery response to simulated microgravity

**Fig. 4:**
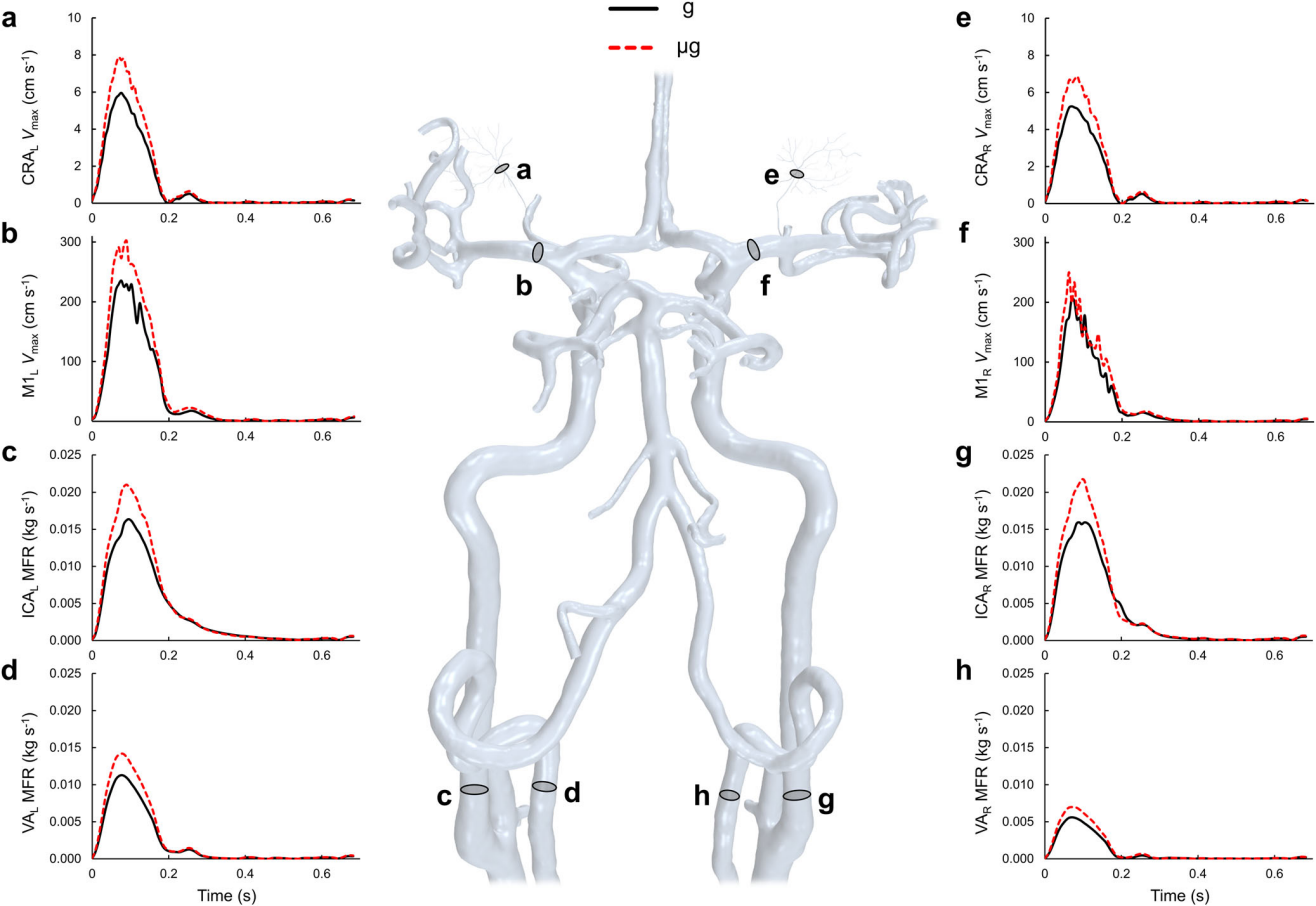
Blood flow waveform changes throughout the cerebrovasculature and the eye. Waveforms are presented for Earth gravity (g; solid black line) and simulated microgravity (µg; dashed red line). Maximal velocity (*V*_max_) in the left (**a**) and right (**e**) central retinal arteries (CRA) and left (**b**) and right (**f**) M1 segment of the middle cerebral artery, as well as mass flow rate waveforms (MFR) in the left (**c**) and right (**g**) internal carotid (ICA_L_ and ICA_R_) and left (**d**) and right (**h**) vertebral (VA_L_ and VA_R_) arteries over the final cardiac cycle are presented.

In response to a 20% increase in cardiac output at the aortic root in the case of simulated microgravity, the computed velocities and flow rates within the cerebrovasculature were observed to increase (Fig. 4). A summary of waveform metrics are presented in Table 2. We found increases in systolic and average mass flow rates under simulated microgravity conditions compared to gravity conditions. Within the M1 segments of the middle cerebral arteries, peak and average maximal velocity were observed to increase in response to simulated microgravity, preferentially on the left side. Average maximal velocity was 34% and 42% greater in the left compared to the right M1 segment in the gravity and simulated microgravity cases respectively. The CRA observed similar increases in peak and average maximal velocity in response to simulated microgravity. In general, the average velocity leading to the retinal arterioles was 9–10% greater in the left compared to the right eye across both gravity and simulated microgravity cases. Given the fixed CRA cross-section, we extracted average volumetric flow rates (average ± standard deviation) leading to the eyes, where we found the peak and average retinal blood flow increased by 32% (57.7 ± 4.1 μl min^-1^ vs. 43.8 ± 2.8 μl min^-1^) and 31% (9.9 ± 0.4 μl min^–1^ vs 7.6 ± 0.3 μl min^–1^), respectively, in the simulated microgravity case compared to the gravity case.

**Table 2:** Different calculated flow and velocity metrics extracted from the gravity (g) and simulated microgravity (µg) cases, as well as the relative change (g → µg) from gravity to simulated microgravity.

Waveform metric	g	µg	% Change (g → µg)
ICA_L_ MFR_peak_ (kg s^-1^)	0.0164	0.0210	28%
ICA_R_ MFR_peak_ (kg s^-1^)	0.0159	0.0217	36%
VA_L_ MFR_peak_ (kg s^-1^)	0.0113	0.0142	26%
VA_R_ MFR_peak_ (kg s^-1^)	0.0056	0.0070	25%
ICA_L_ MFR_ave_ (kg s^-1^)	0.0037	0.0045	21%
ICA_R_ MFR_ave_ (kg s^-1^)	0.0035	0.0042	21%
VA_L_ MFR_ave_ (kg s^-1^)	0.0021	0.0027	25%
VA_R_ MFR_ave_ (kg s^-1^)	0.0010	0.0013	28%
M1_L_ *V*_max, peak_ (cm s^-1^)	235.49	301.57	28%
M1_R_ *V*_max, peak_ (cm s^-1^)	207.03	250.24	21%
CRA_L_ *V*_max, peak_ (cm s^-1^)	5.96	7.88	32%
CRA_R_ *V*_max, peak_ (cm s^-1^)	5.24	6.86	31%
M1_L_ *V*_max, ave_ (cm s^-1^)	41.97	53.58	28%
M1_R_ *V*_max, ave_ (cm s^-1^)	31.33	37.64	20%
CRA_L_ *V*_max, ave_ (cm s^-1^)	1.00	1.31	31%
CRA_R_ *V*_max, ave_ (cm s^-1^)	0.92	1.19	30%
CRA_L_ *Q*_peak_ (µl min^-1^)	45.78	60.60	32%
CRA_R_ *Q*_peak_ (µl min^-1^)	41.83	54.76	31%
CRA_L_ *Q*_ave_ (µl min^-1^)	7.77	10.20	31%
CRA_R_ *Q*_ave_ (µl min^-1^)	7.41	9.61	30%

Peak (MFR_peak_) and average (MFR_ave_) mass flow in the left (ICA_L_) and right (ICA_R_) internal carotid and left (VA_L_) and right (VA_R_) vertebral arteries as well as peak (*V*_max, peak_) and average (*V*_max, ave_) velocity in the M1 segment of the left (M1_L_) and right (M1_R_) middle cerebral artery and left (CRA_L_) and right (CRA_R_) central retinal arteries are presented. Peak (*Q*_peak_) and average (*Q*_ave_) volume flow rates are also provided for the left and right central retinal arteries.

### Retinal vasculature response to simulated microgravity

**Fig. 5:**
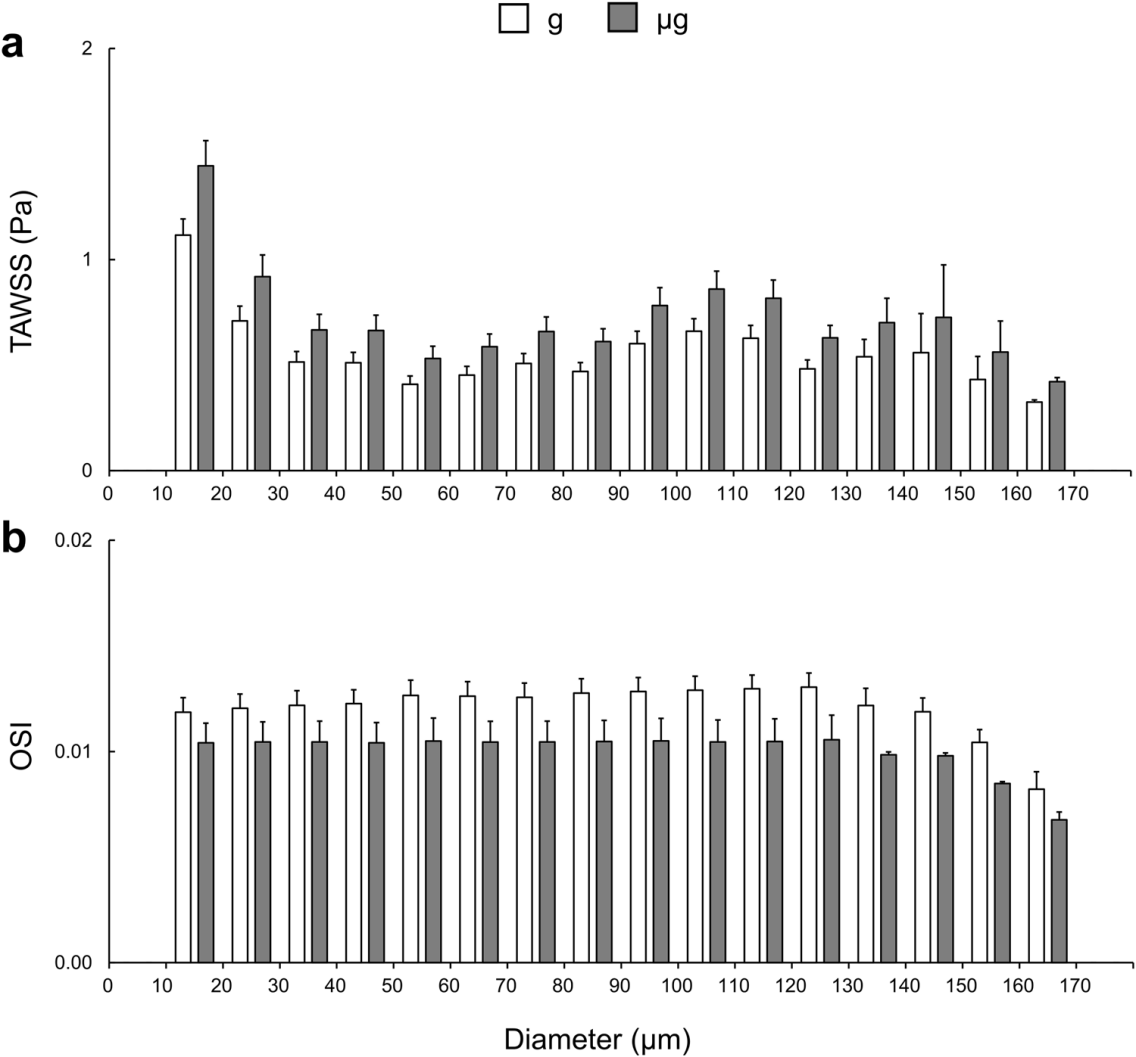
Haemodynamic metrics within the retinal arterioles. Diameter distributed surface averaged haemodynamic metrics for the Earth gravity (g; white) and simulated microgravity (µg; grey) cases. Measures of surface averaged time-averaged wall shear stress (TAWSS) (**a**) and oscillatory shear index (OSI) (**b**) are presented as a combined mean of the left and right eye diameter bins, with error bars representing the corresponding standard deviation of each bin average.

We calculated the mean of surface averaged haemodynamic metrics across the left and right retinal arterioles, which were distributed by corresponding vessel diameter (Fig. 5). In general, TAWSS was found to be greatest in the smallest arterioles (g: 0.71–1.12 Pa and μg: 0.92–1.44 Pa across 10–20 μm diameters), followed by in the larger diameter vessels (g: 0.60–0.66 Pa and μg: 0.78–0.86 Pa across 90–110 μm diameters). Relative to the gravity case, TAWSS increased uniformly across all diameter bands in response to simulated microgravity (29-30%). OSI was almost uniformly distributed across small to large arterioles (g: 0.012–0.013 and μg: 0.010–0.011 across 10–130 μm diameters). The oscillatory shear decreased with increasing diameter in the larger arteriole vessels above 140 μm, irrespective of exposure condition. OSI also decreased in response to simulated microgravity across all diameter bands (–12–19%).

## Discussion

Since the beginning of spaceflight, there has been interest in understanding the effects of space travel on the human body. While the implications of microgravity on muscle mass and strength have been investigated in both animal models^49^ and humans^50^, the effect on blood flow and arterial biomechanics is less understood. Some studies have examined blood flow stasis and thrombosis using ultrasound and revealed important flow abnormalities during microgravity^51^. Others have used computational modelling to replicate the haemodynamic effects of pressure changes and weightlessness^52^. In this study, we investigated the effects of simulated microgravity on vascular biomechanics in a large three-dimensional model of the arterial system, contiguous from the heart through to the eye.

To achieve this, we combined 3D models from different imaging modality data to develop a large 3D model indicative of an arterial blood flow network, similar to methods used previously for 3D geometries spanning from the lower limbs to the CoW^11,15^. We developed a simulation framework for CFD analysis with continuous physical fluid characteristics and fractal tree and resistance outlets which were applied to this large 3D geometry. We then implemented the rudimentary effects of simulated microgravity in the arterial system. This work serves as an interesting proof of concept for future research that may seek to investigate the effects of physiological stimuli on large interconnected arterial networks.

There is considerable interest in the reported vision loss associated with SANS due to prolonged spaceflight. Computer simulations may help reveal some of the biomechanics that may be contributing to the development of SANS^53^. Salerni et al.^10^ constructed a 0D model of the cerebrovasculature, retinal and choroidal vessels, incorporating the effects of changes in aqueous humour and cerebrospinal fluid flow, compression of the lamina cribrosa and the osmosis of fluid at the blood-brain barrier (BBB) in response to simulated microgravity. Although the focus of their study was the investigation of different oncotic pressures and their influence on intraocular and transmural pressures, they found that the parallel configuration of the retina and choroid chambers resulted in increases in normalised retinal flow of approximately 5% for the simulated microgravity case with a weakened BBB, while parallel flow in the choroid and the ciliary body gradually decreased. Although our simulation did not incorporate the effects of intraocular, oncotic or intracranial pressures, we did find that the increase in cardiac output and change in gravitational field in simulated microgravity conditions increased retinal blood flow relative to upright Earth gravity conditions. Within the eye, similar changes to those calculated in our simulations have also been observed during spaceflight. Using colour Doppler ultrasound, Sirek et al.^54^ measured changes in peak systolic velocity in the CRA before, during and after spaceflight. From a database of 14 astronauts, they found an average increase in velocity of 36.1% combined across the left and right eyes from pre-flight values to inflight values, similar to the increases in CRA peak velocity calculated in our study (30-31%). The slightly lower relative changes in velocities in our study may be explained by the assumption of rigid geometry, as Sirek et al. also observed an 11% increase in optic nerve sheath diameter, which may compress the CRA, decreasing its diameter resulting in increases in velocity^55^; this was not accounted for in our model. Interestingly, ground-based experiments have found even greater increases in retinal blood flow, with Laurie et al.^56^ measuring CRA velocity increases of 43–48% in HDT and HDT with hypercapnia compared to seated measures.

An interesting question that arises from these findings is what elevated flow, and therefore shear stress, may mean in the context of the retina and conditions such as SANS. Elevation of shear stress to 2 Pa in bovine retinal endothelial cells has been previously shown to significantly increase retinal endothelium permeability by up to a factor of 14^57^. Higher shear stress conditions (> 1 Pa), as opposed to low-moderate shear (0.1–0.5 Pa), have also been associated with pro-inflammatory responses and barrier dysfunction in human retinal endothelial cells^58^. Higher vascular permeability may not necessarily pose a risk to osmotic balance at the vessel wall provided albumin transport is matched^59^, however, albumin concentration has been found to be significantly lower in astronauts^60,61^. Evidence of retinal endothelial cell dysfunction has been observed previously in mice flown on the international space station (ISS), which exhibited significantly higher retinal endothelial cell apoptosis compared to both Earth controls, as well as to mice that also flew on the ISS while in a centrifuged habitat that produced an effective 1 g of artificial gravity^62^. Our results show that an assumed increase in cardiac output of 20% due to emulating the increase in pre-load from increased venous return^42,46^ during simulated microgravity may result in up to a 30% increase in WSS in the retina, and where higher shear stress is primarily distributed in the smaller arterioles. Although an assumed value of 20% was used in this study^40,41,46^, long-duration spaceflight between 3–6 months has reported the possibility of greater increases in cardiac outputs between 35-41%^42^, with some estimates as high or in excess of 50%^1,43^. As discussed in later sections, if the assumed cardiac waveform in our study is lower than what may be the case for an individual, a higher baseline cardiac output coupled with the corresponding increase in shear stress in the retina attributed to the microgravity environment (which may be a potentially greater increase in cardiac output than the assumed 20%) may predispose these vessels to possible endothelial dysfunction and leakiness, subsequently contributing to the development of oedema in and around the retina. Recent research has postulated a multi-hit hypothesis to the progression of SANS, whereby any oedema caused by endothelial dysfunction may impact the outflow of cerebral spinal fluid which is already impaired in space^63^—in turn contributing to the pressure accumulation on the posterior eye^40^. Nonetheless, given the heterogeneous and non-individual-specific nature of the underlying 3D models used in this study, and the mirroring of retinal vasculature about the left and right sides, these results provide only the initial trends of the shear stress related responses to simulated microgravity in the eye. As such, the remaining pathophysiology of this condition remains highly complex and is likely contributed to by a multitude of factors, each requiring subsequent investigation.

Simulated blood flow to the brain has also been reported by Gallo et al.^4^. In comparison to our findings where blood flow to the cerebrovasculature increased in response to simulated microgravity, they reported general decreases in blood flow in regions throughout the body, such as in the vertebral (–17%) and internal carotid (–19%) arteries. However, their study purposely compared the simulated microgravity results to a reference supine condition. Hence, many of their findings are the inverse to the results in our study, where we observed increases in vertebral and internal carotid artery flows between 21–28%, due to our gravity condition being in an upright reference position. Nevertheless, as suggested by the authors, results of increased flow upon exposure to simulated microgravity would be expected relative to an upright condition^4,64^. Ground-based emulated microgravity research of neck artery flow by Ogoh et al.^65^ measured flow leading to the cerebral vasculature after 57 days of –6 degrees HDT rest as an analogue for prolonged spaceflight. Relative to pre-HDT rest in a supine condition, they observed an average reduction in blood flow in the ICA after 30 (–23%) and 57 days (–15%), while the vertebral arteries remained unchanged, resulting in the vertebral arteries carrying an increased proportion of the cerebrovascular flow relative to pre-HDT. Although we report results relative to an upright condition (and as indicated, our results appear inverted as relative to upright increases compared to relative to supine reductions) we found greater changes in average flow in the vertebral compared to internal carotid arteries, reflecting an increase in proportional cerebrovascular flow in the vertebral arteries between the simulated microgravity and gravity cases.

Interestingly, in general, we also observed higher flows in the left arteries leading to the cerebrovasculature compared to the right, which is also reflected in the flows leading to the eye in the central retinal artery. Although possibly anatomically specific to an individual, this may be due to the left carotid and vertebral arteries originating from branches either closer or directly from the aortic arch, compared to the right side branching from the brachiocephalic artery. Interestingly, naturally higher left-side flow has previously been observed, particularly between vertebral artery sides^66^, which is consistent with the findings of our study in that left vertebral artery flow was substantially higher than that of right vertebral artery flow. Interestingly, though warranting further investigation, additional preferential arterial flow to the left side of the cerebrovasculature may potentially contribute to the findings of additional flow stasis observed in the left jugular vein during spaceflight, which is less pronounced on the right side^67–69^.

Blood flow simulations of isolated 3D geometries leading to, and within the cerebrovasculature, under different gravitational loadings have also been performed previously. Kim et al.^17^ simulated blood flow through compliant carotid bifurcation and CoW 3D geometries, as well as incorporating an autoregulatory mechanism at the arterial outlets, to investigate the changes observed in response to spaceflight. In the carotid artery bifurcation, they found that in order to maintain consistent blood flow to the outlets as per their autoregulation algorithm, the carotid diameter in the simulated microgravity case increased by 6.2% relative to the upright gravity case. As a result, distributions of TAWSS between the cases were observed to decrease almost uniformly under simulated microgravity relative to upright. Similar changes were also observed leading to and within the CoW, with diameter increases in the ICAs (3%), basilar (4.4%) and MCAs (6.9%) under the simulated microgravity case relative to upright. Similar changes in TAWSS were observed with almost uniform decreases in all regions of the CoW and proximal arteries. In comparison, our simulations used a rigid wall boundary condition preventing vessel wall change, and consequently yielded the inverse result, with almost uniform increases in surface averaged TAWSS across the upper body regions of the 3D geometry.

Within the brain itself, MCA velocity changes have also been observed in response to spaceflight microgravity or terrestrial microgravity emulation. In response to HDT and HDT with induced hypercapnia, Laurie et al.^56^ measured increases in average MCA velocity of approximately 20%, an increase similar to the results found in our study (20–28%). In comparison, cerebral blood flow measured in four astronauts after 1 and 2 weeks in space^70^ was found to elucidate non-significant changes in MCA average velocity relative to the pre-flight measurement. However, 1 astronaut did observe a substantial increase in average MCA velocity at the 2-week mark, an increase of approximately 28% relative to pre-flight measurements. The lack of change in MCA velocity could indicate cerebral autoregulation acting for this duration of spaceflight, though this may not necessarily occur across all individuals. Similar non-significant changes in MCA average velocity have also been observed during parabolic flights (15 bouts of 20 s of parabolic freefall), where small average increases (4%) were observed across 16 participants^71^. In comparison to these findings, Iwasaki et al.^72^ found that MCA blood flow velocity in 11 astronauts pre and post-spaceflight (between 3–6 months prior to the flight and within 3 days of landing), for either supine or sitting measurements significantly increased by between 10–13%. The blood flow velocity was then observed to reduce to pre-flight levels after a recovery window of between 1–6 months of landing. This increase in MCA velocity is less substantial than the changes observed in our study, however, this could be attributed to the study subjects returning to Earth for imaging, where the reintroduction of Earth’s gravitational vector may have influenced cerebral blood flow within the first 3 days of landing.

The question remains, however, what these changes in cerebral flows as per the findings of our study, and similarly to others, mean for individuals in microgravity. Although postural changes may result in brain blood flow increasing or decreasing throughout a day within autoregulatory bounds, our results show that simulated microgravity results in a constant increase of 20–30% in brain blood flow, and close to 40% increases in shear stress within the brain. This may have consequences in that increased perfusion of the brain may lead to exacerbated autoregulatory responses resulting in prolonged vessel dilation, reduced myogenic response, which is consistent with mouse spaceflight models^73^, decreased cerebrovascular resistance and consequently any increased pressure acting on the cerebral endothelium could result in oedema^74^. Typical time-averaged shear acting across the sensitive BBB within the cerebrovasculature is in the range of 0.3–3 Pa^75^, and where moderate shear stress within this range has been found to be beneficial to the barrier function of cerebral endothelial cells^76^. However, severely elevated pulsatile shear stress (> 4 Pa) has been associated with the downregulation of BBB tight junction markers, impeding endothelial cell contact^77^. Our findings show that simulated microgravity serves to substantially increase the shear stresses acting both within the cerebrovasculature and retinal vessels. Coupled with additional blood throughput potentially leading to venous stasis and congestion^1,78^, our findings are consistent with causes of fluid oedema associated with exposure to microgravity, which is often observed as a key contributor to the pathogenesis associated with the development of SANS. Nonetheless, future work is required to improve the understanding of the development of SANS as well as the clinical implications of constant elevated flow to the brain.

There is limited data on the effects of microgravity on the coronary arteries, in particular shear stress. We found that TAWSS in the coronary arteries increased in response to simulated microgravity, although both gravity and simulated microgravity case values fell within the normal and atheroprotective shear stress range of 1–7 Pa^79^. Although anecdotal, this finding may support NASA data reporting that, when compared to healthy terrestrially based control populations, astronauts following spaceflight do not have increased differences in cardiovascular and coronary artery disease or standardised mortality^80,81^.

Various in vitro cellular model and in vivo animal model studies have been used to investigate the functional effects of emulated microgravity on endothelial cells and arteries. Hindlimb unloading (HU) is an animal model technique involving the suspension of rodents to create a downwards head tilt and pressure gradient across the body, similar to head-down tilt (HDT) in humans. Despite minimal morphological changes^82^, functional changes such as vasoconstriction and relaxation responses in young HU rat abdominal aorta samples have been found to be reduced relative to control rats^83^. Similar diminished vasoconstriction responses have also been observed in the mesenteric arteries of HU rats^84^ as well as in mice that have flown in space^85^. Alternatively, Shi et al.^86^ found that cultured human umbilical vein endothelial cells experiencing 24 h of emulated microgravity conditions using a clinostat upregulated endothelial nitric oxide synthase, increased cell migration and promoted angiogenic pathways. Similar findings of increased endothelial cell migration and nitric oxide production have been observed by Siamwala et al.^87^ after 2 h of similarly emulated microgravity. Increases in endothelial nitric oxide synthase have also been observed in the aortas of HU mice^88^. In our study, surface averaged TAWSS (1.95–2.52 Pa) across the aorta increased by 17–23% in the simulated microgravity case. Given the mechanoactivation of endothelial nitric oxide synthase is associated with higher shear stresses^89^, haemodynamic responses to simulated or emulated microgravity may induce, on average, somewhat favourable endothelial conditions in larger arteries, such as the aorta, and contribute to any reductions in vasoconstriction.

Blood flow changes in the lower limbs have also been investigated previously from spaceflight data, simulations and ground analogue experiments. Gallo et al.^4^ implemented a large 0D-1D model, combining a 1D arterial tree with 0D representations of circulatory regions and baroreceptor mechanisms to understand the deconditioning of the cardiovascular system during long-duration spaceflight. Compared to upper body flow, they calculated smaller decreases in flow to the lower limb regions of the inner iliac (-2.27%) and femoral (–4.87%) arteries in response to simulated microgravity. Although again, the changes in flow are inverted compared to ours due to using a supine position as their relative condition. Despite this, we also observed a greater proportion of flow distributed to the upper body compared to the lower limbs. After 5 weeks of HDT, a study by Palombo et al.^90^ found that the diameters of the femoral artery were significantly reduced, while non-significant reductions in wall shear rates were measured with ultrasound at the near (–2%) and far (–9%) walls. In our study, we calculated similar small decreases in TAWSS across the iliac arteries (–4%) in response to simulated microgravity, while interestingly the absolute values of TAWSS remained higher compared to the upstream regions such as those in the aorta. Nonetheless, this decrease in shear stress reflects a reduction in blood flow towards the lower limbs in response to simulated microgravity, which is consistent with decreases (though reversible after one month of Earth gravity) in superficial blood flow that has been observed and measured pre and post flight in the lower limbs of astronauts^91^. Prolonged reductions in flow to lower limbs may have implications for the metabolic health in these regions, particularly given the documented musculoskeletal wasting that occurs in space with the reduction in gravitational loading^50,92^. Furthermore, prolonged reductions in perfusion to the lower limbs may present additional risks in the context of the development of peripheral artery conditions or disease, which are generally characterised by reductions in perfusion and ischaemia in these regions^93^. Promising potential countermeasures include lower body negative pressure devices, which serve to counteract upward fluid shift and redistribution by introducing negative pressure about the lower limbs^94^. These devices may also serve as a potential countermeasure for the pathophysiological development of SANS, which is suspected to be caused, at least in part, by this fluid gradient and redistribution of fluid throughout the body^94^.

Despite differences in exact geometry, dimensional representation or environmental conditions, and variation in demographics associated with imaging sources, we observed some similarities between our results and existing large-scale simulation networks. Blanco et al.^95^ developed a 1D anatomically detailed arterial network model consisting of over 2,000 arterial vessels using a 3D circulatory representation as a geometrical substrate. Although the inlet flow rate in their model was greater than the inlet flow rate used in our gravity case, the average flow calculated across the VAs matched our results to within 2%, though the average flow in the ICAs was 25% greater in their study compared to ours. Xiao et al.^11^ developed a simulation of a 3D deformable full-body arterial network consisting of arterial vessels ranging from the tibial artery to the CoW. Using diameters and flow data provided in their work, the average velocity in the left middle cerebral artery was found to be 54% greater than the same artery in our gravity simulation. However, their inlet flow waveform was substantially higher, with a systolic flow rate approximately double that used in our gravity case simulation. Xiao et al. also calculated and presented the shear stress throughout the entire geometry but highlighted that the mesh used was insufficient to ensure grid independence in the WSS fields and that the results were only to provide an indication of capability. Our simulation framework demonstrates that this resolution is achievable to capture the WSS and associated haemodynamic metrics.

Nonetheless, despite serving as an initial proof of concept study for investigating continuously connected arterial networks in response to environments, such as simulated microgravity, the methods proposed in this work are not without limitations and need for future development.

Firstly, as we used a mixture of 3D model data from different imaging modalities and sources across different subjects, the 3D model developed does not represent a single individual. Consequently, the underlying 3D model heterogeneity may influence the absolute data reported, such as the distributions of surface averaged shear stress or the amplitude of velocity waveforms. As such, although absolute data is reported for reference, the findings from this study aimed to focus on the relative changes and trends offered by rudimentary simulation of microgravity, whereby any systematic heterogeneity effects may be nullified through cancellation given the use of the same 3D model in both Earth gravity and simulated microgravity cases. Nonetheless, by incorporating real imaging data, the model does at least represent a continuous human arterial network that is physiologically possible, albeit not singularly subject specific and formed from sources with varying demographics. Future work using the methods and approaches described in this study would ultimately use individual-specific imaging data for the construction of the continuous arterial network 3D model. One key benefit of this approach, however, is that (as demonstrated in our study) retrospective imaging data can be combined to form the 3D arterial network. Consequently, future work with individual astronaut data would potentially be feasible—enabling greater insights into the haemodynamics occurring throughout a large proportion of the arterial cardiovasculature in the spaceflight environment. Alternatively, subject-specific data could be combined to understand how individuals with pre-existing cardiovascular risk factors may be predisposed in environments of varying gravitational load, such as during spaceflight, on Martian or lunar surfaces, or during the acute hyper-gravity associated with planetary exit or re-entry.

Secondly, we used a rigid wall model that did not account for the movement of the arterial wall. Although astronauts undergoing 6-month duration spaceflight have been observed to experience the equivalent of 10–20 years of arterial aging and stiffening^96^, this remains a limitation given vessels are inherently compliant in healthy populations, of which would be the case in astronauts currently undertaking spaceflight missions. Alternatively, wall movement could be achieved in future work using fluid-structure interaction (FSI) modelling. This was not performed, however, in this initial study given the significant additional computational load required for FSI simulation, computational stability and interfacing requirements across such a large arterial domain, and as the arterial wall thicknesses and tissue material properties were either unable to be resolved or known, respectively. Furthermore, in future work that would ideally use individual-specific imaging, obtaining wall thickness and tissue material properties would either be impossible, severely invasive or limited to only the larger arteries. Additionally, these methods would need to account for vessel pretension embedded in the 3D reconstructions from vessel imaging being captured, which represents vessels already at arterial pressure. Despite rigid wall modelling not accounting for the deformation fluctuations experienced throughout the cardiac cycle and instead representing a snapshot in time, comparable distributions of WSS between rigid and FSI simulations have previously been observed, although rigid wall simulations generally overestimate instantaneous WSS compared to FSI^34,97^. Surface and time-averaged metrics, such as those used in this study, have also been observed to be similar between rigid wall and FSI methods^98^.

Thirdly, for all arterial outlets except for the retinal arterioles, we used a mixture of known estimated flow splits to arterial networks and then Murray’s law to estimate the distribution of flow throughout numerous arterial outlet regions. This approach was adopted due to the simplicity of implementation, but it limited the simulations in terms of accounting for any changes in vessel compliance or autoregulatory constrictions/dilations. Alternative outlet modelling approaches that should be considered include using varying power law exponents based on literature or modelling distal resistance and compliance using multi-element Windkessel models, which may enable the incorporation of autoregulatory mechanisms in the cerebrovasculature as well as account for the effects of venous stasis and congestion that are generally associated with the spaceflight environment^1,78^. Additionally, MRI or ultrasound methods could be employed to measure subject-specific regional flow distributions, as opposed to the assumed values as described in Table 1.

Fourthly, the cardiac output of the inlet flow condition adapted from Brown et al.^34^ may be inadequate for the geometry developed, which is reflected in the variation in results for the gravity condition with other large arterial simulations. Future work should aim to use subject-specific measured cardiac output, which could be obtained using MRI or duplex ultrasound methods. Alternatively, in the absence of cardiac output data, a parametrically swept range of different cardiac outputs could be investigated. However, as the goal of this study was to provide an initial framework for comparing relative changes in response to simulated microgravity in a large 3D continuous arterial network, the relative changes were found to be somewhat consistent with emerging microgravity research and measured data. Additionally, while we incorporated the aortic root as part of the geometry, we implemented the flow at the aortic valve surface as a simplified parabolic velocity flow profile with a fixed orifice area, neglecting the natural helical and three-dimensional nature of blood flow ejected from the aortic valve.

Finally, we assumed the flow to be within the laminar flow regime, which is a common approach in arteries outside of the aorta, however, turbulence is likely induced within the ascending aorta due to the high ejection velocities at the aortic valve as well as during the deceleration phase of the cardiac cycle. As a key region of interest in this study were the vessels leading to and within the eye, which are known to exhibit mostly laminar flow, this regime was considered appropriate and any turbulence generated at the aortic root was assumed to have minimal effect on reported haemodynamics in these regions. Modelling using large eddy simulation (LES) may be more appropriate in future studies, particularly to investigate changes in haemodynamics within the aorta and nearby larger arteries, though this was not performed due to the increasingly high computational cost associated with this modelling approach.

In this study, we aimed to demonstrate that large-scale 3D arterial networks can be constructed across a wide range of vessel calibres from 3D models derived from numerous image datasets and that the resulting geometry can be used to understand the change in haemodynamics in response to simulated microgravity. From our simulations, we found similarities with existing spaceflight simulation models and measured data—specifically that blood flow and shear stress decrease towards the lower limbs and increase towards the cerebrovasculature and within the eyes in response to simulated microgravity exposure relative to an upright position in Earth gravity. This framework may also prove useful to simulate the changes in haemodynamics in other equally challenging environments influencing the cardiovascular system.

### Supplementary information

Supplementary Material

## Data Availability

Data not directly presented in the article, such as geometries and simulation files, can be made available on reasonable request to the authors.

## Code availability

The fluid simulation code used in this study is commercially available.

## References

[1] Norsk P (2020). Adaptation of the cardiovascular system to weightlessness: surprises, paradoxes and implications for deep space missions. Acta Physiol..

[2] Vernice NA, Meydan C, Afshinnekoo E, Mason CE (2020). Long-term spaceflight and the cardiovascular system. Precis. Clin. Med..

[3] Gerber B, Singh J-L, Zhang Y, Liou W (2018). A computer simulation of short-term adaptations of cardiovascular hemodynamics in microgravity. Comput. Biol. Med..

[4] Gallo C, Ridolfi L, Scarsoglio S (2020). Cardiovascular deconditioning during long-term spaceflight through multiscale modeling. npj Microgravity.

[5] Xiao N, Alastruey J, Alberto Figueroa C (2014). A systematic comparison between 1-D and 3-D hemodynamics in compliant arterial models. Int. J. Numer. Method Biomed. Eng..

[6] Morris PD (2016). Computational fluid dynamics modelling in cardiovascular medicine. Heart.

[7] Morgan WH, Hazelton ML, Yu DY (2016). Retinal venous pulsation: Expanding our understanding and use of this enigmatic phenomenon. Prog. Retin. Eye Res..

[8] Tang Y, He Y (2018). Numerical modeling of fluid and oxygen exchanges through microcirculation for the assessment of microcirculation alterations caused by type 2 diabetes. Microvasc. Res..

[9] Guidoboni G (2014). Intraocular pressure, blood pressure, and retinal blood flow autoregulation: a mathematical model to clarify their relationship and clinical relevance. Investig. Ophthalmol. Vis. Sci..

[10] Salerni F (2019). Biofluid modeling of the coupled eye-brain system and insights into simulated microgravity conditions. PLoS ONE.

[11] Xiao N, Humphrey JD, Figueroa CA (2013). Multi-scale computational model of three-dimensional hemodynamics within a deformable full-body. Arterial Netw. J. Comput. Phys..

[12] Mukherjee D, Shadden SC (2017). Inertial particle dynamics in large artery flows–Implications for modeling arterial embolisms. J. Biomech..

[13] Mukherjee, D., Jani, N. D., Selvaganesan, K., Weng, C. L. & Shadden, S. C. Computational assessment of the relation between embolism source and embolus distribution to the circle of Willis for improved understanding of stroke etiology. *J. Biomech. Eng*. **138**, 10.1115/1.4033986 (2016). 10.1115/1.4033986 27367268

[14] Gounley J (2019). Computing the ankle-brachial index with parallel computational fluid dynamics. J. Biomech..

[15] Feiger B, Adebiyi A, Randles A (2021). Multiscale modeling of blood flow to assess neurological complications in patients supported by venoarterial extracorporeal membrane oxygenation. Comput. Biol. Med..

[16] Chen M, Shadden SC, Hart JC (2016). Fast coherent particle advection through time-varying unstructured flow datasets. IEEE Trans. Vis. Comput. Graph..

[17] Kim CS, Kiris C, Kwak D, David T (2005). Numerical simulation of local blood flow in the carotid and cerebral arteries under altered gravity. J. Biomech. Eng..

[18] Rebhan J, Parker LP, Kelsey LJ, Chen FK, Doyle BJ (2019). A computational framework to investigate retinal haemodynamics and tissue stress. Biomech. Model. Mechanobiol..

[19] Thomas HJ (2020). Assessment of cerebrovascular responses to physiological stimuli in identical twins using multimodal imaging and computational fluid dynamics. J. Appl. Physiol..

[20] Majeed K (2021). Coronary (18)F-sodium fluoride PET detects high-risk plaque features on optical coherence tomography and CT-angiography in patients with acute coronary syndrome. Atherosclerosis.

[21] Kelsey LJ (2021). Low endothelial shear stress is associated with high-risk coronary plaque features and microcalcification activity. JACC Cardiovasc. Imaging.

[22] Sakalihasan, N. et al. Tissue PET vascular metabolic imaging and peripheral plasma biomarkers in the evolution of chronic aortic dissections. *Eur. Heart J. Cardiovasc. Imaging* **16**, 626–633 (2015). 10.1093/ehjci/jeu283 25588800

[23] Budai A, Bock R, Maier A, Hornegger J, Michelson G (2013). Robust vessel segmentation in fundus images. Int. J. Biomed. Imaging.

[24] Dorner GT (2002). Calculation of the diameter of the central retinal artery from noninvasive measurements in humans. Curr. Eye Res..

[25] Lang J, Kageyama I (1990). The ophthalmic artery and its branches, measurements and clinical importance. Surg. Radiol. Anat..

[26] Drobnjak D (2017). Relationship between retinal vessel diameters and retinopathy in the Inter99 Eye Study. Clin. Transl. Endocrinol..

[27] Roache P (1994). Perspective: a method for uniform reporting of grid refinement studies. J. Fluids Eng..

[28] Schwer, L. E. IS YOUR MESH REFINED ENOUGH? Estimating Discretization Error using GCI. *German LS-DYNA Forum* (2008).

[29] Kelsey LJ, Powell JT, Norman PE, Miller K, Doyle BJ (2017). A comparison of hemodynamic metrics and intraluminal thrombus burden in a common iliac artery aneurysm. Int. J. Numer. Methods Biomed. Eng..

[30] Liu D (2009). Computational analysis of oxygen transport in the retinal arterial network. Curr. Eye Res..

[31] Malek J (2015). Computational analysis of blood flow in the retinal arteries and veins using fundus image. Comput. Math. Appl..

[32] Reinke W, Johnson PC, Gaehtgens P (1986). Effect of shear rate variation on apparent viscosity of human blood in tubes of 29 to 94 microns diameter. Circ. Res..

[33] Karimi S (2014). Effect of rheological models on the hemodynamics within human aorta: CFD study on CT image-based geometry. J. Non-Newton. Fluid Mech..

[34] Brown AG (2012). Accuracy vs. computational time: translating aortic simulations to the clinic. J. Biomech..

[35] Olufsen MS (1999). Structured tree outflow condition for blood flow in larger systemic arteries. Am. J. Physiol. Heart Circ. Physiol..

[36] Wang Q (2011). Imaging retinal capillaries using ultrahigh-resolution optical coherence tomography and adaptive optics. Invest. Ophthalmol. Vis. Sci..

[37] Perdikaris P, Grinberg L, Karniadakis GE (2015). An effective fractal-tree closure model for simulating blood flow in large arterial networks. Ann. Biomed. Eng..

[38] Torok A, Gallagher M, Lasbareilles C, Ferrè ER (2019). Getting ready for Mars: How the brain perceives new simulated gravitational environments. Q. J. Exp. Physiol..

[39] Garrett-Bakelman FE (2019). The NASA twins study: a multidimensional analysis of a year-long human spaceflight. Science.

[40] Zwart SR (2017). Astronaut ophthalmic syndrome. FASEB J..

[41] Norsk P (2006). Vasorelaxation in space. Hypertension.

[42] Norsk P, Asmar A, Damgaard M, Christensen NJ (2015). Fluid shifts, vasodilatation and ambulatory blood pressure reduction during long duration spaceflight. J. Physiol..

[43] Hughson RL, Peterson SD, Yee NJ, Greaves DK (2017). Cardiac output by pulse contour analysis does not match the increase measured by rebreathing during human spaceflight. J. Appl. Physiol..

[44] White RJ, Blomqvist CG (1998). Central venous pressure and cardiac function during spaceflight. J. Appl. Physiol..

[45] Liu Z (2015). Alterations in the heart rate and activity rhythms of three orbital astronauts on a space mission. Life Sci. Space Res.

[46] Krämer BK, Mang JF, Schubert R (2018). The effect of microgravity on central aortic blood pressure. Am. J. Hypertens..

[47] Watenpaugh DE (2001). Effects of spaceflight on human calf hemodynamics. J. Appl. Physiol..

[48] Navasiolava N (2020). Vascular and microvascular dysfunction induced by microgravity and its analogs in humans: mechanisms and countermeasures. Front. Physiol..

[49] Okada R (2021). Transcriptome analysis of gravitational effects on mouse skeletal muscles under microgravity and artificial 1 g onboard environment. Sci. Rep..

[50] Edgerton VR (1995). Human fiber size and enzymatic properties after 5 and 11 days of spaceflight. J. Appl. Physiol..

[51] Marshall-Goebel K (2019). Assessment of jugular venous blood flow stasis and thrombosis during spaceflight. JAMA Netw. Open.

[52] Mohammadyari P, Gadda G, Taibi A (2021). Modelling physiology of haemodynamic adaptation in short-term microgravity exposure and orthostatic stress on Earth. Sci. Rep..

[53] Stenger, M. B. & Tarver, W. J. *Evidence Report: Risk of Spaceflight Associated Neuro-ocular Syndrome (SANS)*. *Human Research Program, Human Health Countermeasures*. (National Aeronautics and Space Administration, 2017).

[54] Sirek AS (2014). Doppler ultrasound of the central retinal artery in microgravity. Aviat. Space Environ. Med..

[55] Hughson RL, Irving EL (2021). Spaceflight not an eye-popping experience for astronauts. J. Physiol..

[56] Laurie SS (2017). Effects of short-term mild hypercapnia during head-down tilt on intracranial pressure and ocular structures in healthy human subjects. Physiol. Rep..

[57] Lakshminarayanan S, Gardner TW, Tarbell JM (2000). Effect of shear stress on the hydraulic conductivity of cultured bovine retinal microvascular endothelial cell monolayers. Curr. Eye Res..

[58] Molins B (2019). Shear stress modulates inner blood retinal barrier phenotype. Exp. Eye Res..

[59] Tarbell JM (2010). Shear stress and the endothelial transport barrier. Cardiovasc. Res..

[60] Cirillo M (2003). Low urinary albumin excretion in astronauts during space missions. Nephron. Physiol..

[61] Zwart SR, Auñón-Chancellor SM, Heer M, Melin MM, Smith SM (2022). Albumin, oral contraceptives, and venous thromboembolism risk in astronauts. J. Appl. Physiol..

[62] Mao, X. W. et al. Impact of spaceflight and artificial gravity on the mouse retina: biochemical and proteomic analysis. *Int. J. Mol. Sci*. **19**, 10.3390/ijms19092546 (2018). 10.3390/ijms19092546 PMC6165321 30154332

[63] Barisano G (2022). The effect of prolonged spaceflight on cerebrospinal fluid and perivascular spaces of astronauts and cosmonauts. Proc. Natl Acad. Sci. USA.

[64] Zhang L-F, Hargens AR (2018). Spaceflight-induced intracranial hypertension and visual impairment: pathophysiology and countermeasures. Physiol. Rev..

[65] Ogoh S, Sato K, de Abreu S, Denise P, Normand H (2020). Arterial and venous cerebral blood flow responses to long-term head-down bed rest in male volunteers. Exp. Physiol..

[66] Bogren HG, Buonocore MH, Gu WZ (1994). Carotid and vertebral artery blood flow in left- and right-handed healthy subjects measured with MR. Veloc. Mapp. J. Magn. Reson. Imaging.

[67] Pavela J (2022). Surveillance for jugular venous thrombosis in astronauts. Vasc. Med..

[68] Arbeille P, Provost R, Zuj K, Vincent N (2015). Measurements of jugular, portal, femoral, and calf vein cross-sectional area for the assessment of venous blood redistribution with long duration spaceflight (vessel imaging experiment). Eur. J. Appl. Physiol..

[69] Arbeille P (2001). Adaptation of the left heart, cerebral and femoral arteries, and jugular and femoral veins during short- and long-term head-down tilt and spaceflights. Eur. J. Appl. Physiol..

[70] Iwasaki KI (2007). Human cerebral autoregulation before, during and after spaceflight. J. Physiol..

[71] Klein T (2019). The influence of microgravity on cerebral blood flow and electrocortical activity. Exp. Brain Res..

[72] Iwasaki KI (2021). Long-duration spaceflight alters estimated intracranial pressure and cerebral blood velocity. J. Physiol..

[73] Taylor CR (2013). Spaceflight-induced alterations in cerebral artery vasoconstrictor, mechanical, and structural properties: implications for elevated cerebral perfusion and intracranial pressure. FASEB J..

[74] Cipolla, M. J. *The Cerebral Circulation* (Morgan & Claypool Life Sciences, 2009). 21452434

[75] Wong AD (2013). The blood-brain barrier: an engineering perspective. Front. Neuroeng..

[76] Cucullo L, Hossain M, Puvenna V, Marchi N, Janigro D (2011). The role of shear stress in blood-brain barrier endothelial physiology. BMC Neurosci..

[77] Garcia-Polite F (2017). Pulsatility and high shear stress deteriorate barrier phenotype in brain microvascular endothelium. J. Cereb. Blood Flow. Metab..

[78] Rosenberg MJ (2021). Comparison of dural venous sinus volumes before and after flight in astronauts with and without spaceflight-associated neuro-ocular syndrome. JAMA Netw. Open.

[79] Gijsen F (2019). Expert recommendations on the assessment of wall shear stress in human coronary arteries: existing methodologies, technical considerations, and clinical applications. Eur. Heart J..

[80] Ade CJ, Broxterman RM, Charvat JM, Barstow TJ (2017). Incidence rate of cardiovascular disease end points in the National Aeronautics and Space Administration Astronaut Corps. J. Am. Heart Assoc..

[81] Elgart SR (2018). Radiation exposure and mortality from cardiovascular disease and cancer in early NASA astronauts. Sci. Rep..

[82] Wilkerson MK, Muller-Delp J, Colleran PN, Delp MD (1999). Effects of hindlimb unloading on rat cerebral, splenic, and mesenteric resistance artery morphology. J. Appl. Physiol..

[83] Delp MD, Brown M, Laughlin MH, Hasser EM (1995). Rat aortic vasoreactivity is altered by old age and hindlimb unloading. J. Appl. Physiol..

[84] Behnke BJ, Zawieja DC, Gashev AA, Ray CA, Delp MD (2008). Diminished mesenteric vaso- and venoconstriction and elevated plasma ANP and BNP with simulated microgravity. J. Appl. Physiol..

[85] Behnke BJ (2013). Effects of spaceflight and ground recovery on mesenteric artery and vein constrictor properties in mice. FASEB J..

[86] Shi F (2012). Effects of simulated microgravity on human umbilical vein endothelial cell angiogenesis and role of the PI3K-Akt-eNOS signal pathway. PLoS ONE.

[87] Siamwala JH (2010). Simulated microgravity perturbs actin polymerization to promote nitric oxide-associated migration in human immortalized Eahy926 cells. Protoplasma.

[88] Grenon SM, Jeanne M, Aguado-Zuniga J, Conte MS, Hughes-Fulford M (2013). Effects of gravitational mechanical unloading in endothelial cells: association between caveolins, inflammation and adhesion molecules. Sci. Rep..

[89] Urschel K, Tauchi M, Achenbach S, Dietel B (2021). Investigation of wall shear stress in cardiovascular research and in clinical practice-from bench to bedside. Int. J. Mol. Sci..

[90] Palombo C (2015). Large artery remodeling and dynamics following simulated microgravity by prolonged head-down tilt bed rest in humans. Biomed. Res. Int..

[91] Ishihara A (2020). Blood flow in astronauts on Earth after long space stay. Acta Astronaut..

[92] Vandenburgh H, Chromiak J, Shansky J, Del Tatto M, Lemaire J (1999). Space travel directly induces skeletal muscle atrophy. FASEB J..

[93] Natarajan B, Patel P, Mukherjee A (2020). Acute lower limb ischemia-etiology. Pathol., Manag. Int. J. Angiol..

[94] Ly V, Velichala SR, Hargens AR (2022). Cardiovascular, lymphatic, and ocular health in space. Life.

[95] Blanco PJ, Watanabe SM, Passos MARF, Lemos PA, Feijóo RA (2015). An anatomically detailed arterial network model for one-dimensional computational hemodynamics. IEEE Trans. Biomed. Eng..

[96] Hughson RL (2016). Increased postflight carotid artery stiffness and inflight insulin resistance resulting from 6-mo spaceflight in male and female astronauts. Am. J. Physiol. Heart Circ. Physiol..

[97] Reymond P, Crosetto P, Deparis S, Quarteroni A, Stergiopulos N (2013). Physiological simulation of blood flow in the aorta: Comparison of hemodynamic indices as predicted by 3-D FSI, 3-D rigid wall and 1-D models. Med. Eng. Phys..

[98] Lantz J, Karlsson M (2012). Large eddy simulation of LDL surface concentration in a subject specific human aorta. J. Biomech..

[99] Steele BN, Olufsen MS, Taylor CA (2007). Fractal network model for simulating abdominal and lower extremity blood flow during resting and exercise conditions. Comput. Methods Biomech. Biomed. Eng. Imaging Vis..

[100] Sato K, Ogoh S, Hirasawa A, Oue A, Sadamoto T (2011). The distribution of blood flow in the carotid and vertebral arteries during dynamic exercise in humans. J. Physiol..

[101] Ramanathan T, Skinner H (2005). Coronary blood flow. BJA Educ..

[102] Ambarki K (2013). Blood flow of ophthalmic artery in healthy individuals determined by phase-contrast magnetic resonance imaging. Invest. Ophthalmol. Vis. Sci..

