# Supplementary material for: Modelling large scale artery haemodynamics from the heart to the eye in response to simulated microgravity
Source: NPJ Microgravity. 2024 Jan 13;10:7. doi: 10.1038/s41526-024-00348-w (PMC10787773; doi:10.1038/s41526-024-00348-w)
Supplement: Supplementary file 1 — Supplementary Material [file 41526_2024_348_MOESM1_ESM.pdf]

MODELLING LARGE SCALE ARTERY HAEMODYNAMICS FROM THE HEART TO THE EYE IN RESPONSE TO SIMULATED MICROGRAVITY

HARRISON T. CADDY[1,2]

LACHLAN J. KELSEY[1,3]

LOUIS P. PARKER[4]

DANIEL J. GREEN[2]

BARRY J. DOYLE[1,3]

[1]*Vascular Engineering Laboratory*, Harry Perkins Institute of Medical Research, Queen Elizabeth II Medical Centre, Nedlands, Australia and the UWA Centre for Medical Research, The University of Western Australia, Perth, Australia

[2]*School of Human Sciences (Exercise and Sport Sciences)*, The University of Western Australia, Perth, Australia

[3]*School of Engineering*, The University of Western Australia, Perth, Australia

[4]*FLOW, Department of Engineering Mechanics*, KTH Royal Institute of Technology, Stockholm, Sweden

SHORT TITLE: Heart to eye haemodynamic response to simulated microgravity

AUTHOR FOR CORRESPONDENCE:

Associate Professor Barry J. Doyle

barry.doyle@uwa.edu.au

Supplementary Tables

Supplementary Table 1 Variable meshing parameters and mesh sizes used for the coarse, medium and fine meshes.

**Meshing Parameter**	**Coarse Mesh**	**Medium Mesh**	**Fine Mesh**
Number of Prism Layers	2	4	5
Prism Layer Stretching Ratio	2.000	1.260	1.189
Boundary Growth Rate	1	2	3
Number of Elements	19,614,806	29,856,210	43,247,920

Supplementary Table 2 Constant meshing parameters used for the surface and prism layer meshers respectively.

**Meshing Parameter**	**Value (mm)**
Target Surface Mesh Size	0.200
Minimum Surface Mesh Size	0.002
Prism Layer Thickness	1.000
Prism Layer Thickness (Cerebrovasculature)	0.170
Prism Layer Thickness (Coronary)	0.170
Prism Layer Thickness (Retinal)	0.013
Prism Layer Thickness (Carotid/Subclavian)	0.350

Supplementary Table 3 GCI results for the different CFD metrics of average and maximum wall shear stress (WSS) across the entire geometry, as well as mass flow rates (MFR) for the left (ICA_L) and right (ICA_R) internal carotid, left (VA_L) and right (VA_R) vertebral and left (CRA_L) and right (CRA_R) central retinal arteries respectively. CFD metrics were obtained at steady systole flow conditions.

**CFD Metric**	**GCI**
Average WSS (Pa)	0.10%
Max WSS (Pa)	1.95%
MFR ICA_L (kg/s)	2.59%
MFR VA_L (kg/s)	0.01%
MFR ICA_R (kg/s)	0.05%
MFR VA_R (kg/s)	0.22%
MFR CRA_L (kg/s)	0.12%
MFR CRA_R (kg/s)	0.09%
